# High Nucleotide Diversity Accompanies Differential DNA Methylation in Naturally Diverging Populations

**DOI:** 10.1093/molbev/msad068

**Published:** 2023-03-22

**Authors:** James Ord, Toni I Gossmann, Irene Adrian-Kalchhauser

**Affiliations:** Institute for Fish and Wildlife Health, University of Bern, Bern, Switzerland; Faculty of Biological and Environmental Sciences, University of Helsinki, Helsinki, Finland; Computational Systems Biology, Faculty of Biochemical and Chemical Engineering, TU Dortmund University, Dortmund, Germany; Institute for Fish and Wildlife Health, University of Bern, Bern, Switzerland

**Keywords:** DNA methylation, stickleback, epigenetic, nucleotide diversity, local adaptation

## Abstract

Epigenetic mechanisms such as DNA methylation (DNAme) are thought to comprise an invaluable adaptive toolkit in the early stages of local adaptation, especially when genetic diversity is constrained. However, the link between genetic diversity and DNAme has been scarcely examined in natural populations, despite its potential to shed light on the evolutionary forces acting on methylation state. Here, we analyzed reduced-representation bisulfite sequencing and whole-genome pool-seq data from marine and freshwater stickleback populations to examine the relationship between DNAme variation (between- and within-population) and nucleotide diversity in the context of freshwater adaptation. We find that sites that are differentially methylated between populations have higher underlying standing genetic variation, with diversity higher among sites that gained methylation in freshwater than those that lost it. Strikingly, although nucleotide diversity is generally lower in the freshwater population as expected from a population bottleneck, this is not the case for sites that lost methylation, which instead have elevated nucleotide diversity in freshwater compared with marine. Subsequently, we show that nucleotide diversity is higher among sites with ancestrally variable methylation and also positively correlates with the sensitivity to environmentally induced methylation change. The results suggest that as selection on the control of methylation state becomes relaxed, so too does selection against mutations at the sites themselves. Increased epigenetic variance in a population is therefore likely to precede genetic diversification.

## Introduction

DNA methylation (DNAme) is an epigenetic mark whose roles in genome regulation, including gene expression regulation and transposable element suppression, have been well studied (He et al. 2011). Its role in local adaptation and long-term evolution, however, for example, via plasticity, remains a topic of active debate. Methylome data support a potential role for DNAme in local adaptation in several species (Dubin et al. 2015; Sammarco et al. 2022), revealing that some genomic regions show differential methylation (DM) between different locally adapted populations. Populations (Johnson and Kelly 2020) and species (Vernaz et al. 2021) with low genetic divergence from one another have been found to differ considerably in DNAme patterns at environmentally relevant loci, raising the possibility that DNAme can be a source of phenotypic variation that increases adaptive potential of populations when genetic diversity is challenged (Flores et al. 2013). Although such variation in DNAme can be environmentally induced or stochastic (Richards 2006), it is also influenced by genetic diversity. For example, DNAme is determined by the presence of sites with the capacity to be methylated (typically in a CpG context) and is subject to *trans*- and *cis*-regulation (Villicaña and Bell 2021). Therefore, the potential for methylation and corresponding plasticity is determined by the local genomic context, and thus the “epigenetic potential” of a population evolves at the sequence level (Kilvitis et al. 2017; see also Adrian-Kalchhauser et al. 2020).

It is also established that the epigenetic conformation of the genome affects the propensity for sequence change. Notably, DNAme can influence mutation rates due to the higher susceptibility of methylated Cs to spontaneous deamination to form thymine (Xia et al. 2012; Poulos et al. 2017; Zhou et al. 2020). In mammals, CpG mutation rates have been estimated at 10–50× higher than in other sequence contexts (Walser and Furano 2010). CpG mutation rate also has a nuanced relationship with methylation levels, as CpG sites with the highest mutation rates in human populations were observed to have low-to-intermediate methylation levels in cultured cells (Xia et al 2012). Therefore, by influencing sequence evolution, epigenetic variation may have unappreciated roles in the emergence of genomic novelties and adaptations (Storz et al. 2019; Guerrero-Bosagna 2020), as well as mediating environmental influences on sequence evolution (Guerrero-Bosagna 2012; Lu et al. 2021).

Despite the interdependence of DNAme and sequence variation, the potential importance of this link in local adaptation has been largely overlooked. For example, typical workflows for detection of DM tend to exclude CpG sites that are not detected at a certain coverage in a certain proportion of individuals (e.g., Akalin et al. 2012), and therefore it could be assumed that genetic diversity of those sites is irrelevant. However, these sites may nevertheless harbor genetic variants in the population, the relative frequencies of which may be informative about evolutionary forces acting on methylation state, potentially allowing further dissection of the manner in which DM evolves in the context of local adaptation. Indeed, methylation sites within certain promoters have already been shown to exhibit selective sweep signatures in *Arabidopsis* (Shirai et al. 2021). Epigenetic diversification is one of many possible routes to local adaptation (e.g., Smith et al. 2016) but may occur in conjunction with others, such as selection on discrete new mutations (hard sweeps) or on standing genetic variation (soft sweeps) (Bernatchez 2016; Hermisson and Pennings 2017). For example, if epigenetic modifications at multiple loci could confer similar adaptive benefit, epigenetic diversification could occur in conjunction with a soft sweep. Furthermore, given the heightened mutation rate of methylated Cs and its complex relationship with methylation levels (Xia et al 2012), acquisition of methylation in a divergent population or a change in methylation level may influence mutation rates at affected sites. There is therefore a need to examine the relationships between DM and nucleotide diversity in divergent populations.

The three-spined stickleback fish (*Gasterosteus aculeatus*) has long been a popular model to study the genetics and, more recently, the epigenetics of local adaptation. Ancestrally, a marine fish, *G. aculeatus*, has repeatedly and rapidly colonized freshwater (FW) habitats over the last few millennia, with large waves of colonizations having occurred since the formation of glacial lakes following the last ice age (Jones et al. 2012; Roberts Kingman et al. 2021). FW-adapted morphs show numerous phenotypic adaptations including the loss of armor plating (Barrett et al. 2008) and changes to kidney morphology (Hasan et al. 2017). Phenotypic and genetic divergence has been observed over short timescales (Lescak et al. 2015), with large shifts in frequencies of particular alleles having been observed over just a few years in newly established lake populations (Roberts Kingman et al. 2021). Such rapid fixation of alleles on the basis of standing genetic variation is characteristic of a soft sweep (Bernatchez 2016). However, the rapid adaptability and high plasticity of sticklebacks (Day et al. 1994) also make the contribution of epigenetic variation to FW adaptation compelling. Multiple studies have used bisulfite sequencing (BS-seq) to reveal differentially methylated sites in CpG context (DMCs) or differentially methylated regions (DMRs) between marine and FW populations. Some DMCs and DMRs are in the vicinity of genes relevant to FW adaptation (Smith et al. 2015; Artemov et al. 2017; Heckwolf et al. 2020; Hu et al. 2021).

A potential role for epigenetic variation in FW adaptation is especially pertinent given that the formation of FW populations has been characterized by population bottlenecks, constraining genetic diversity. Steep declines in the effective population size *Ne* have been observed in newly established FW populations from both time series experiments (Aguirre et al. 2022) and ancient DNA (Kirch et al. 2021). Interestingly, in their comparison of gill DNA methylomes between marine and FW fish in the White Sea region, Artemov et al. (2017) showed that FW fish had higher variance of DNAme compared with marine fish, in line with the idea that higher epigenetic variation could compensate for reduced genetic diversity, enhancing the adaptive potential of a population following a bottleneck.

Although genome and/or epigenome data have been generated from multiple stickleback populations across the Northern Hemisphere, the White Sea population complex is unique in that a variety of different data types have been generated from populations inhabiting the same region, including DNAme (Artemov et al. 2017), mRNA and small RNAseq (Rastorguev et al. 2016; Rastorguev et al. 2017), and whole-genome pool-seq (Terekhanova et al. 2014, 2019). FW colonization in this region has occurred relatively recently, with the oldest sampled lake estimated to have been formed ∼700 years ago. Although nucleotide diversity is typically lower in FW populations, likely due to past bottlenecks (Terekhanova et al. 2014), patterns of nucleotide diversity at methylation sites, and DMCs specifically, have not been addressed. Shifts in nucleotide diversity at DMCs may be informative about the evolutionary forces acting on DNAme during local adaptation, namely, the tightening or loosening of selective constraint on methylation state in a new environment.

Here, we combine epigenetic and genetic data from the White Sea stickleback population complex to study the interactions between methylation differences and nucleotide diversity during FW colonization. We examined nucleotide diversity in relation to methylation divergence, variance, and the environmental inducibility of methylation state, considering both variance and inducibility as indicators of the relative stringency DNAme regulation.

## Results

### Elevated Nucleotide Diversity Accompanies DM but Level Depends on the Direction of Methylation Change

**Fig. 1.**
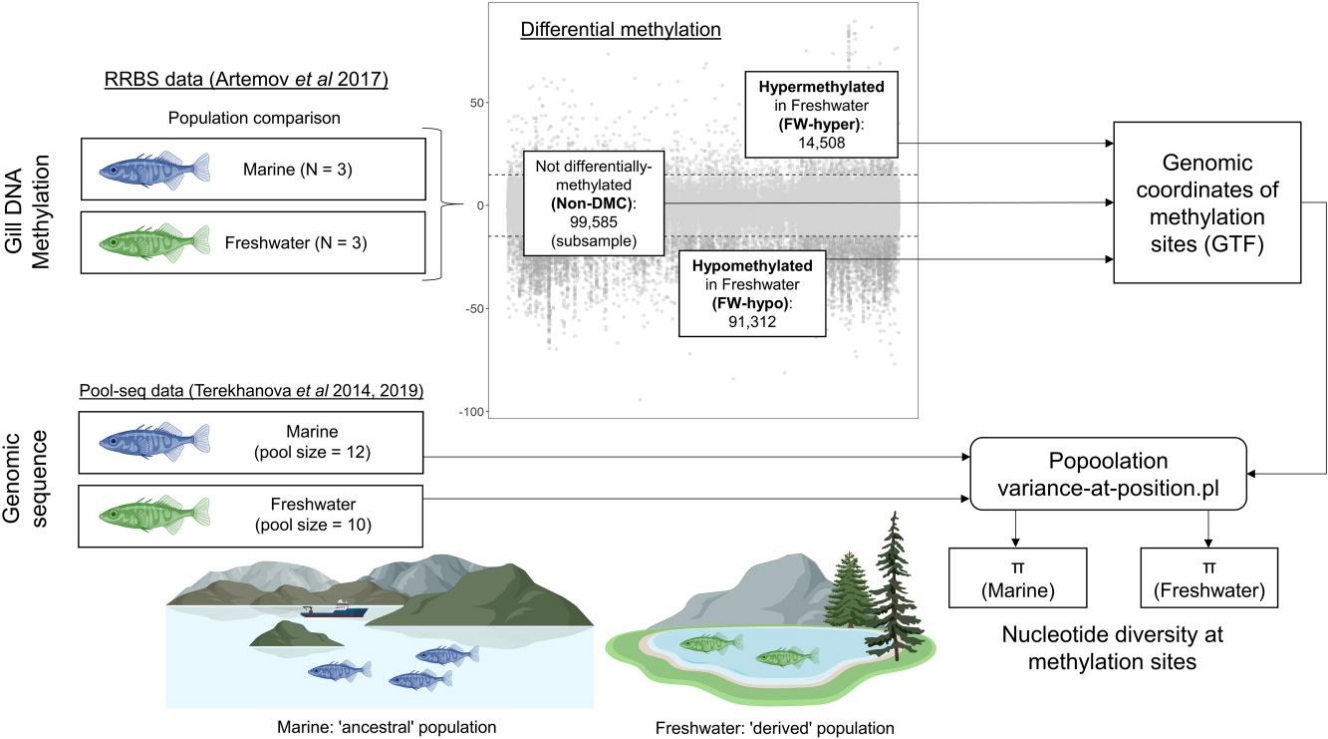
Analysis workflow for obtaining nucleotide diversity estimates for differentially methylated sites. Site-level DM analysis was performed to compare marine (considered as “ancestral” population) versus FW sticklebacks (considered as “derived” population) using gill RRBS data previously published by Artemov et al. (2017). Taking the marine population as the reference methylation state, sites were classified as not differentially methylated (non-DMC; no significant difference in percentage of methylated copies between populations), FW-hypo (significantly lower percentage of methylated copies in FW compared to marine), or FW-hyper (significantly higher percentage of methylated copies in FW compared with marine). For non-DMCs, a subset of the total was used, comprising ∼11% of the total non-DMCs (see Materials and Methods). Coordinates of sites belonging to the three site classes (non-DMC, FW-hypo, and FW-hyper) were compiled in a GTF file for use with the variance-at-position.pl script from the Popoolation toolkit. The nucleotide diversity (*π*) of each site class on each chromosome was estimated from whole-genome pool-seq data (Terekhanova et al. 2014, 2019) of marine and FW fish derived from the same or similar geographic locations as those taken for the RRBS data.

For generation of DNAme and genome sequence data, respectively, both Artemov et al. and Terekhanova et al. sampled FW fish from the same lake (Lake Mashinnoye) and marine fish from nearby coastal locations in the Kandalaksha Gulf. A combined analysis of samples from these two data sets therefore allowed us to identify differentially methylated sites in CpG context (DMCs) between marine and FW populations and examine the nucleotide diversity of these sites in separate samples of those populations (fig. 1). The reduced-representation bisulfite sequencing (RRBS) data (Artemov et al. 2017) were derived from gill tissue from a total of 11 individuals. These included six individuals as part of the main population comparison (*n* = 3 per population) and a further five experimental treatment animals that were used for a subsequent analysis of site inducibility. After filtering to remove sites with C-T/G-A single nucleotide polymorphisms (SNPs) detected in RRBS individuals, which could otherwise lead to spurious counts of unmethylated Cs, the analysis included just over 1 million CpG sites with at least 5× alignment coverage in all individuals, comprising ∼6.9% of CpGs in the genome. The pool-seq data (Terekhanova et al. 2014, 2019) comprised sequenced material of two pools containing 12 marine and ten FW individuals and with genome coverage (high-quality alignments) of 10.4× and 8×, respectively.

We first derived measures of nucleotide diversity of differentially methylated (DMCs) and nondifferentially methylated sites in CpG context (non-DMCs) in the form of *π* (average number of pairwise differences within population) (Nei and Li 1979), Watterson's *θ* (population-scaled mutation rate) (Watterson 1975), and Tajima's *D* (Tajima 1989). *π* and *θ* are complementary measures of within-population nucleotide diversity, while Tajima's *D* is a composite statistic derived from these two measures and can identify deviation of a sequence from neutrality. We also derived pairwise Fst (ratio of between-to-within population diversity; Weir and Cockerham 1984), which in a local sequence context can signify differential selection between populations. All nucleotide diversity measures were derived from pool-seq data at methylation sites detected from RRBS data (fig. 1). Considering non-DMCs as representing a baseline level of nucleotide diversity at cytosines in CpG context, we compared the *π* of non-DMCs with that of DMCs that either lost methylation (at least 15% fewer methylated copies at *P* ≤ 0.05, hypomethylated in FW [FW-hypo]) or gained methylation in FW compared with marine (at least 15% more methylated copies at *P* </= 0.05, hypermethylated in FW [FW-hyper]). Substantially more FW-hypo sites (91,320) were detected than FW-hyper sites (14,508), while 895,121 sites were non-DMCs. For nucleotide diversity analyses, we subsampled non-DMCs by taking one random non-DMC within 2 Mb of each DMC, resulting in a subsample of 99,585, ∼11% of the total number. For each of these categories, nucleotide diversity measures were calculated per chromosome.

**Fig. 2.**
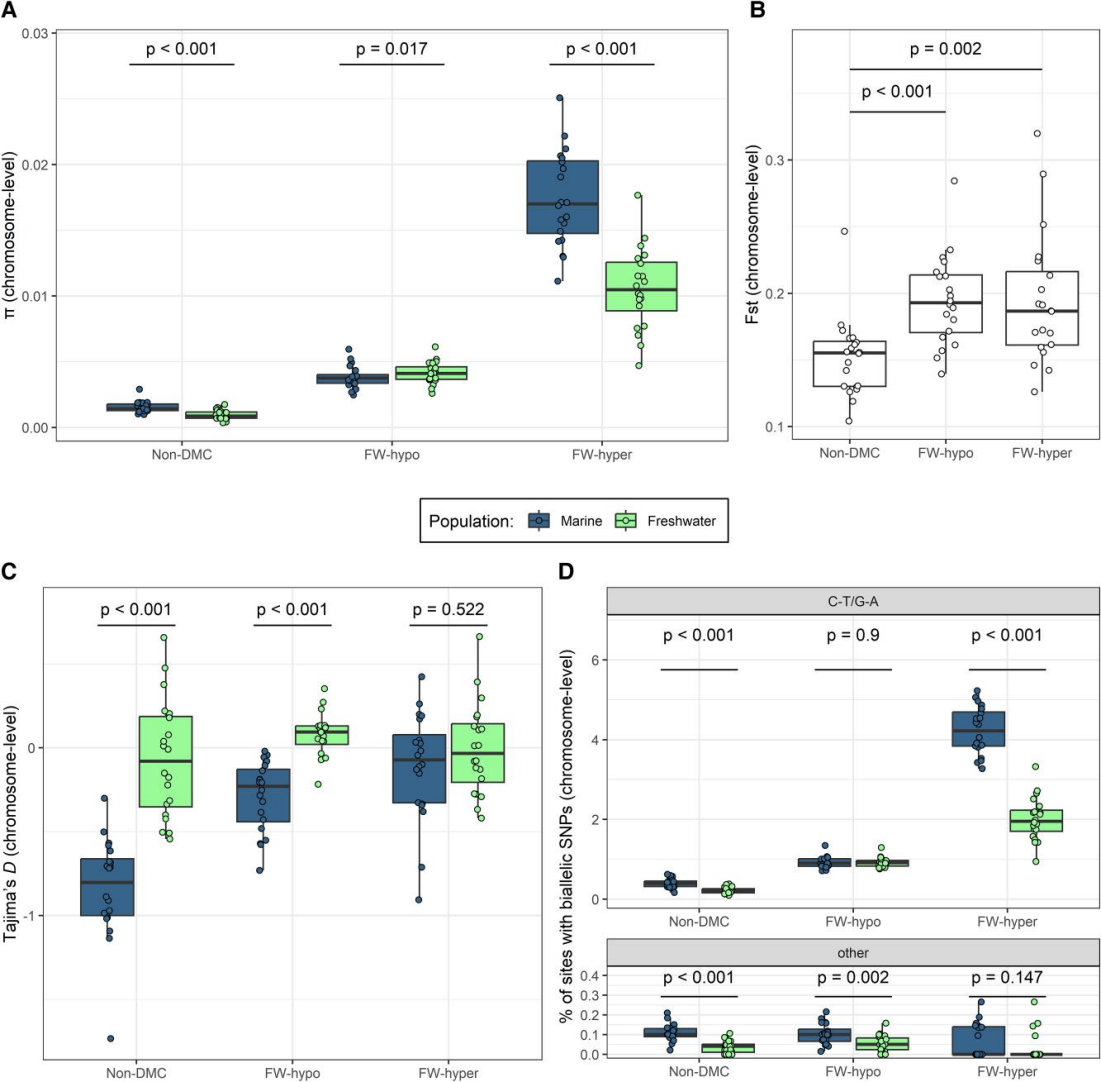
Nucleotide diversity of differentially methylated sites in CpG context. (*A*) *π* (average number of pairwise differences), (*B*) Fst (marine vs. FW), and (*C*) Tajima's *D* estimated from pool-seq of marine and FW sticklebacks for three classes of methylation site identified from RRBS individuals and classified according to the direction of methylation difference in FW fish compared with marine (nondifferentially methylated [non-DMC], FW-hypo, or FW-hyper). (*D*) Percentage of sites in each site class harboring biallelic SNPs of the type C-T/G-A (upper panel) or other types (C-A/G-T or C-G/G-T, lower panel), estimated separately for each chromosome. Average numbers of sites per chromosome were 4979, 4566, and 725 for non-DMC, FW-hypo, and FW-hyper, respectively. All estimates (*π*, Fst, Tajima's *D*, and percentage of C-T/G-A SNPs) are per chromosome. *P*-values derive from paired Wilcoxon tests (*A*, *C*, and *D*) or paired *t*-tests (*B*) (comparison of chromosome pairs).

We observed consistent associations between DM and nucleotide diversity. DMCs showed elevated *π* compared with non-DMCs regardless of population, with the highest *π* observed among sites that gained methylation in FW (FW-hyper) (fig. 2*A*). When comparing *π* between populations for each category of sites, *π* was slightly though significantly reduced in FW compared with marine at non-DMCs (paired Wilcoxon test, *P* < 0.001), reflecting the expected reduction in nucleotide diversity in the derived population. *π* calculated for sliding windows across chromosome 1 suggested that the pattern of reduced diversity in FW is a genome-wide feature (supplementary fig. S1*A*, Supplementary Material online) that likely resulted from a past bottleneck. FW *π* was similarly lower at FW-hyper sites (*P* < 0.001) but not at FW-hypo sites that rather showed elevated *π* in FW compared with marine (*P* = 0.017). Watterson's *θ* showed a similar pattern to that of *π*, although there was no significant difference between marine and FW at FW-hypo sites (supplementary fig. S2, Supplementary Material online). Between-population diversity (Fst) showed significant differences between DMCs and non-DMCs, with both FW-hypo and FW-hyper sites showing significantly higher Fst values than non-DMCs (paired *t*-tests, *P* < 0.01 in both cases; fig. 2*B*). Tajima's *D* was mostly negative in both populations as indicated by sliding windows across chromosome 1 (supplementary fig. S1*B*, Supplementary Material online) but was higher in FW, consistent with the scenario of a population bottleneck following FW colonization. Tajima's *D* of methylation sites largely reflected this chromosome-wide pattern of higher values in FW, with the exception of FW-hyper sites that showed no significant difference in Tajima's *D* between the two populations (fig. 2*C*). Tajima's *D* tended to be higher among DMCs than non-DMCs in marine but not in FW.

These patterns of elevated nucleotide diversity were not driven by enrichment for DMCs in regions of high diversity. Rather, elevated *π* of DMCs was found to be strongly localized around individual DMCs (supplementary fig. S3, Supplementary Material online). The pattern was largely consistent across different genomic features including CpG islands, gene bodies, promoters, and intergenic regions (supplementary fig. S3, Supplementary Material online). No clear pattern of localized elevated diversity was observed for DMCs that fell within DMRs (supplementary fig. S3, Supplementary Material online); however, only a fraction of FW-hypo (∼17%) and FW-hyper DMCs (∼12%) fell within DMRs.

Next, we determined which mutation types were most likely to be driving the elevated *π* of DMCs and specifically whether this was driven by an overabundance of C-T transitions. To this end, the percentages of sites in each category harboring biallelic SNPs of different types (C-T/G-A, C-A/G-T, or C-G/G-C) were calculated from the pool-seq data. The majority of SNPs were C-T/G-A, comprising 90% of SNPs across all categories in marine and 94% in FW. The proportion of sites harboring biallelic C-T/G-A SNPs across the three categories of methylation site and two populations showed a similar pattern to that of *π*, with FW-hyper sites harboring the highest proportion of C-T/G-A SNPs in both populations (fig. 2*D*). Marine had more C-T/G-A SNPs than FW in the non-DMC (paired Wilcoxon test, *P* < 0.001) and FW-hyper categories (*P* < 0.001) but not the FW-hypo category, in which FW and marine had similar proportions of C-T/G-A SNPs. Meanwhile, the percentage of other SNP types showed no clear differences between the site categories. Therefore, the generally increased nucleotide diversity among DMCs relative to non-DMCs seemed to be driven by a greater occurrence of C-T mutations.

### Higher Nucleotide Diversity of Infrequently Methylated DMCs

**Fig. 3.**
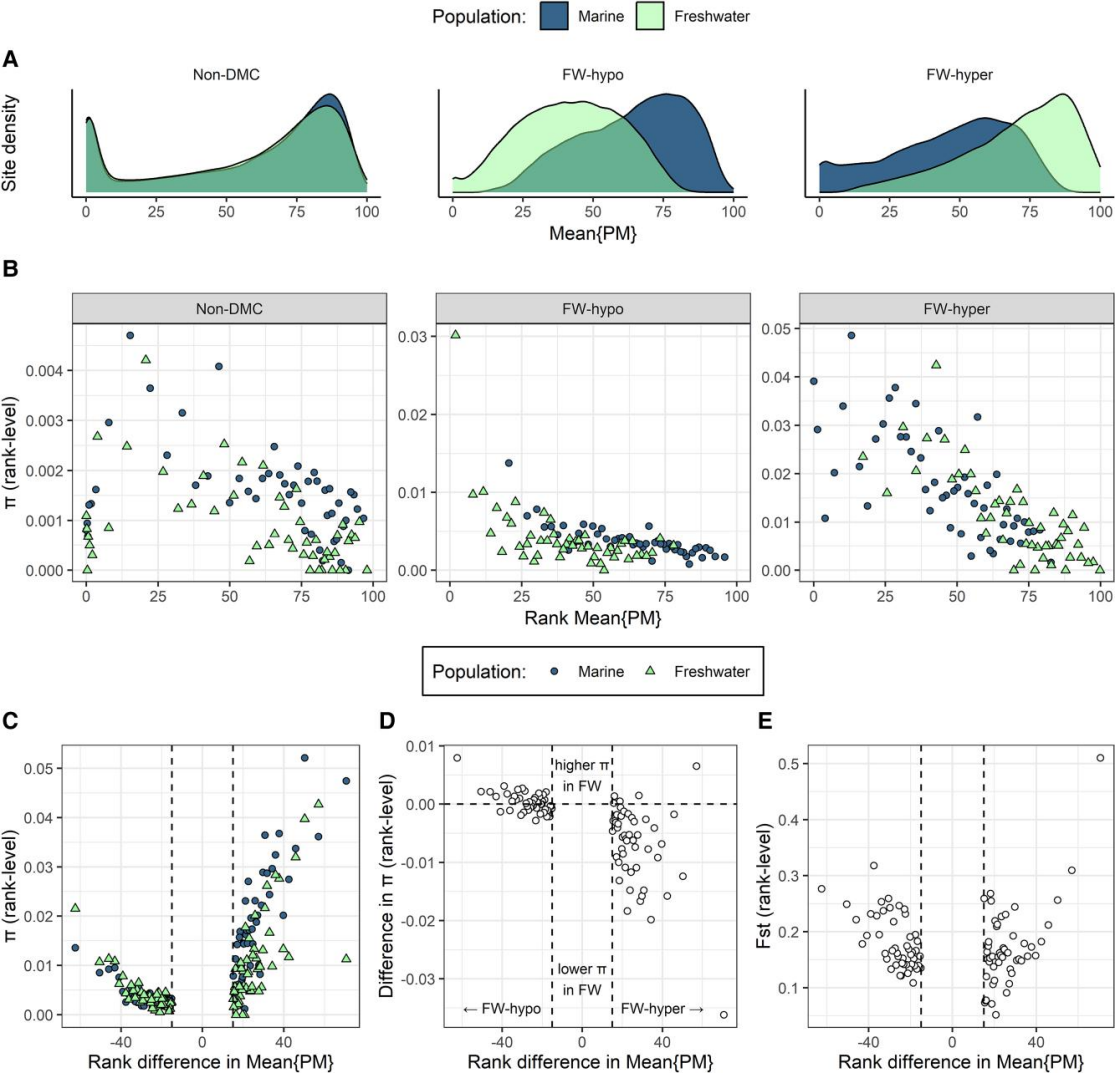
Distribution of mean percentage of methylation (percentage of methylated copies at a given CpG site; mean{PM}) in three site categories and their relationship with nucleotide diversity in marine and FW populations. (*A*) The relative distribution of sites across values of mean{PM} in each population and site category. (*B*) *π* of sites that were ranked according to mean{PM} in three site categories. A single *π* estimate was derived for all the sites in a given rank, and π is plotted against the mean{PM} of the rank. Sites were ranked separately for marine (circles) and FW (triangles) populations and for non-DMC (left panel), FW-hypo (middle panel), and FW-hyper sites (right panel). (*C***–***E*) Nucleotide diversity (*C*), difference in nucleotide diversity (*π* of FW − *π* of marine) (*D*), and pairwise Fst (marine vs. FW) (*E*) of sites that were ranked according to difference in mean{PM} (mean{PM} of FW − mean{PM} of marine). For each population, a single *π* estimate was derived for all the sites in a given rank. In (*B*–*E*), 50 ranks are included (per population in the case of *B* and *C*). Each rank contains an average of 2012, 1826, and 290 sites for non-DMC, FW-hypo, and FW-hyper, respectively.

The finding that sites that gained methylation in FW (FW-hyper) had the highest *π* and highest proportion of C-T/G-A mutations in marine (fig. 2) was contrary to expectations, as these sites would be expected to be infrequently methylated in marine and therefore not at high risk of mutation via deamination. We therefore tested the relationship between *π* and the distributions of mean percentage of methylation (hereafter mean{PM}) across the three site categories. Here, mean{PM} refers to the average percentage of copies on which the C is methylated or in other words the average frequency of the methylation mark among copies of given CpG site. We found that non-DMCs displayed a bimodal density distribution, with most sites either very frequently (>75%) or very infrequently methylated (fig. 3*A*, left). Meanwhile, the distributions of mean{PM} of DMCs were markedly different to those of non-DMCs. FW-hypo sites were characterized by a shift from mostly high mean{PM} in marine to mostly intermediate mean{PM} in FW (fig. 3*A*, middle). Mirroring this pattern, FW-hyper sites were characterized by a shift from low–intermediate mean{PM} in marine to high mean{PM} in FW (fig. 3*A*, right).

Because pool-seq data are not appropriate for estimating *π* at the level of a single site, we used a ranking procedure to examine the relationship between mean{PM} and *π*. Sites were divided into ranks according to their mean{PM}, with higher ranks containing sites with higher mean{PM}. This ranking was performed separately for each population and each site category and a measure of *π* obtained for each rank. The relationship between the rank-level mean{PM} and *π* was clearly nonmonotonic for non-DMCs, with the highest values appearing at low-to-intermediate mean{PM} of ∼25% (fig. 3*B*, left). The higher *π* of FW-hypo sites in FW appeared to be driven largely by sites in the low–intermediate range (fig. 3*B*, middle). Among FW-hyper sites, those with the highest mean{PM} clearly contributed to the lower *π* of these sites in FW (fig. 3*B*, right).

We also examined the relationship of *π* with the population difference in mean{PM} (i.e., the extent of hypomethylation or hypermethylation). We observed that among FW-hypo sites, the FW population had the largest increases in the *π* where the hypomethylation was strongest (fig. 3*C* and *D*). FW-hyper sites also increased in *π* with the extent of hypermethylation (fig. 3*C*), but this also corresponded with greater loss of *π* in FW (fig. 3*D*). Meanwhile, sites with larger difference in mean{PM} in either direction (methylation loss or gain) had higher Fst (fig. 3*E*).

### High Nucleotide Diversity Accompanies High Variability in Ancestral Methylation

**Fig. 4.**
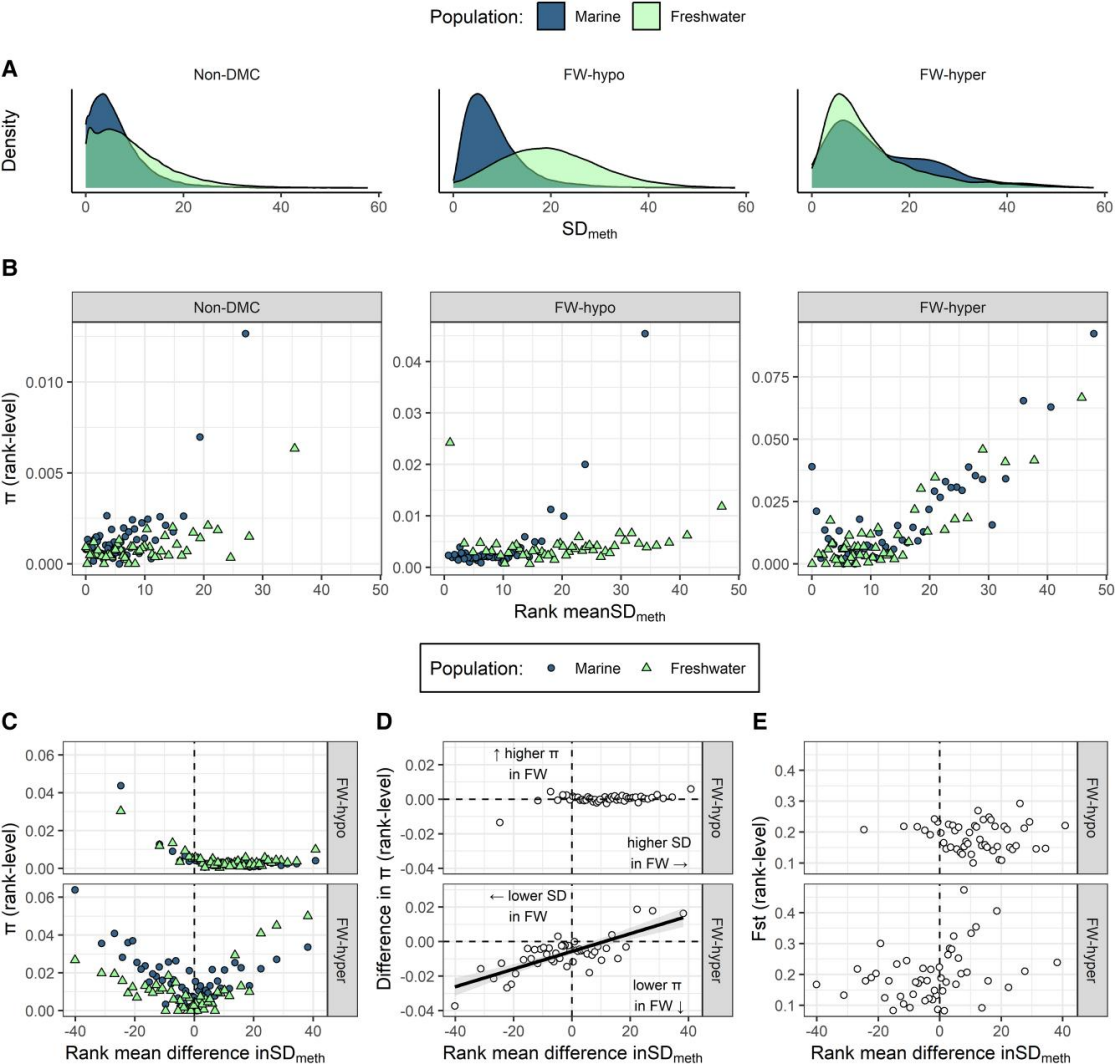
Distribution of standard deviation of percentage of methylation levels (SD_meth_) in three site categories and two populations and their relationship with nucleotide diversity. (*A*) The relative distributions of SD_meth_ in each population and category. (*B*) *π* of sites that were ranked according to their SD_meth_. A single *π* estimate was derived for all the sites in a given rank, and this rank-level *π* is plotted against the mean SD_meth_ of sites in the rank. Sites were ranked separately for marine (circles) and freshwater (triangles) populations and for non-DMC (left panel), FW-hypo (middle panel), and FW-hyper sites (right panel). (*C*–*E*) *π* (*C*), difference in *π* (*π* of FW − *π* of marine) (*D*), and pairwise Fst (marine vs. FW) (*E*) of sites that were ranked according to difference in SD_meth_ between marine and FW (SD_meth_ of FW − SD_meth_ of marine), where higher values represent higher SD_meth_ in FW and lower values represent lower SD_meth_ in FW. For each population, a single *π* estimate was derived for all the sites in a given rank. In (*B*–*E*), 50 ranks are included. Each rank contains an average of 2012, 1826, and 290 sites for non-DMC, FW-hypo, and FW-hyper, respectively. Trend line in (*D*) derived from a linear model and ribbon shows SEM.

Considering that sites with intermediate mean{PM} are liable to have more variable methylation frequency than those with very low or very high mean{PM}, we also considered the relationship between *π* and the standard deviation of percentage methylation (hereafter SD_meth_). We predicted that sites with more variable methylation would have higher nucleotide diversity, reasoning that the methylation state of these sites is not stringently controlled and therefore mutations at these sites may have little impact on function. We first examined the distributions of SD_meth_ values of sites in the non-DMC, FW-hypo, and FW-hyper categories. Non-DMCs were largely invariable, with slightly more variable methylation in FW compared with marine (fig. 4*A*, left), consistent with the observations of Artemov et al. (2017). FW-hypo sites were characterized by a pronounced increase in SD_meth_ from ancestral to derived population, shifting from a left-skewed distribution in marine to a Gaussian-like distribution in FW (fig. 4*A*, middle). Meanwhile, FW-hyper sites showed a skewed distribution in marine with an elongated plateau to the right, while methylation was less variable in FW (fig. 4*A*, right).

To examine the relationship between *π* and the variability in methylation, sites were ranked according to SD_meth_ in each population and site category. We observed that *π* increased steeply at an SD_meth_ above ∼15 in all three site categories in the marine population and two of the three site categories in FW (fig. 4*B*). For the *π* of FW-hypo sites in FW, however, there was no obvious relationship with the exception of two ranks showing highly elevated *π* at opposite ends of the range of SD_meth_ values (fig. 4*B*, middle). The high *π* of the lowest rank, which contradicted the trend observed in the other categories, is likely attributable to high ancestral diversity of sites that have almost completely lost methylation in FW (and therefore attain very low variance) but retain high ancestral nucleotide diversity. The relationship between *π* and the shift in SD_meth_ would appear to support this notion because sites with the largest decrease in SD_meth_ in FW also have the highest *π* in marine (fig. 4*C*). Shifts in the SD_meth_ were also associated with shifts in *π* (fig. 4*D*). At both FW-hypo and FW-hyper sites, there was a trend towards reduced *π* with decreased SD_meth_ and increased *π* with increased SD_meth_. For FW-hypo sites, this was only apparent at extreme shifts in SD_meth_, while for FW-hyper sites, there was a significant linear relationship (linear model, *R*^2^ = 0.58, *P* < 0.001). No clear relationships were observed between the shift in SD_meth_ and Fst for either FW-hypo or FW-hyper categories (fig. 4*E*).

### Environmentally Inducible DNAme Is Linked with Higher Nucleotide Diversity

**Fig. 5.**
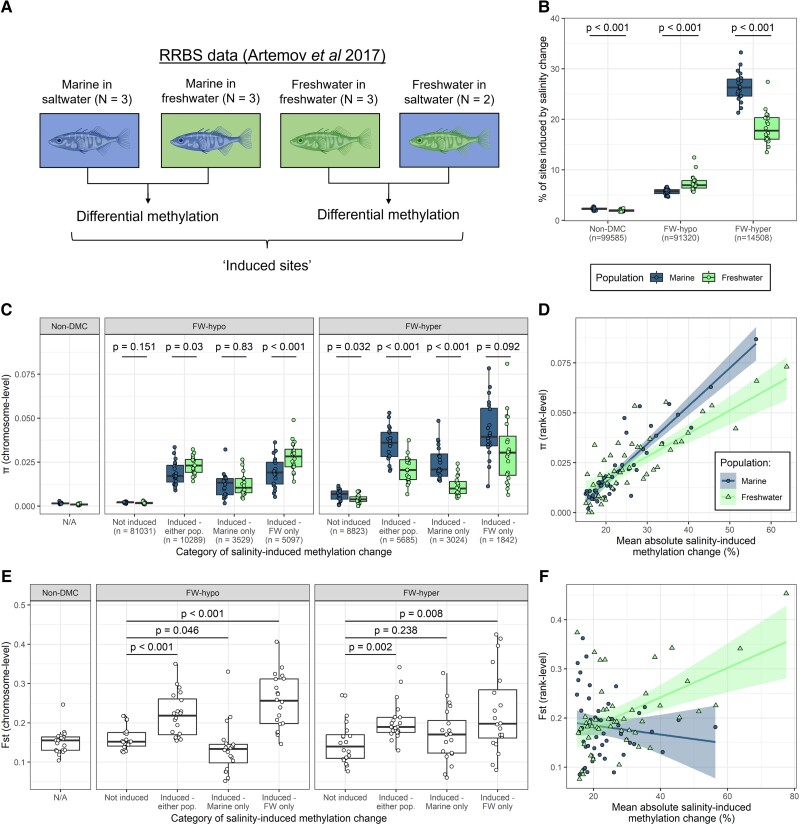
Nucleotide diversity of differentially methylated sites in relation to their capacity for induced methylation change in response to environmental salinity. (*A*) Additional RRBS data deriving from experimental salinity treatments performed by Artemov et al (2017) (marine fish placed in FW and FW fish placed in saltwater) were used to identify sites that were inducible in response to salinity change in either of the two populations. (*B*) Percentage of sites in the non-DMC, FW-hypo, and FW-hyper categories that were induced in response to salinity change in the marine and FW populations. *P*-values derived from paired Wilcoxon tests. (*C*) Per-chromosome estimates of *π* for FW-hypo and FW-hyper sites divided according their capacity for induced gill methylation change in response to a change in environmental salinity, considering sites that were induced in neither of the populations, either of the two populations, or only in one of the two populations (marine or FW). *π* of non-DMCs is shown in separate panel for comparison. *P*-values derived from paired *t*-tests. (*D*) *π* of inducible DMCs that were ranked according to their mean absolute induced change in percentage of methylation (i.e., regardless of the direction). Within each population, only sites that were significantly differentially methylated in response to salinity (mean difference in percentage of methylation ≥15 or ≤15, *P* < 0.05) were considered. Separate ranks were obtained for marine and FW and a single *π* estimate obtained for each rank. (*E*) Per-chromosome estimates of pairwise Fst (FW vs. marine) of FW-hypo and FW-hyper sites divided according to their capacity for induced methylation change. Fst of non-DMCs is shown in separate panel for comparison. *P*-values derived from paired *t*-tests. (*F*) Pairwise Fst of inducible DMCs that were ranked according to their mean absolute induced change in percentage of methylation (i.e., regardless of the direction of the change). For (*D*) and (*F*), 50 ranks were used for marine and 49 for FW. Each rank contains an average of 227 sites for marine and 228 sites for FW. Trend lines derived from linear models and ribbons show SEM.

So far, we have considered DM in regard to losses or gains of methylation that have been detected in a population ∼700 years after its colonization of a new environment. Although such differences may result from evolution, differences in methylation state can also be directly induced by the environment. Such inducibility may be not only important for adaptation but also subject to genetic variation. We therefore analyzed additional samples from the data set by Artemov et al. (2017), as in addition to the main population comparison, the authors also quantified gill methylation differences in fish from each population in response to a change in environmental salinity. Fish from each population were exposed to the opposite conditions, with marine fish exposed to reduced salinity and FW fish exposed to increased salinity (fig. 5*A*). Here, we reasoned that environmental inducibility of methylation state could also reflect the degree of genetic versus environmental control of methylation state, similar to increased SD_meth_ possibly reflecting relaxed control of methylation state. Sites whose methylation state is more responsive to the environment could be assumed to be under looser genetic control and possibly relaxed selection. In total, sites that were induced in either population constituted 3.8% of non-DMCs, 11.3% of FW-hypo, and 39.2% of FW-hyper sites, with DMCs, and particularly FW-hyper sites therefore being enriched for induced sites. When considering the proportions of induced sites in each population separately (fig. 5*B*), FW had a higher proportion of induced sites than marine among FW-hypo sites and a lower proportion among FW-hyper sites, a pattern that almost perfectly mirrored that which was observed for *π* (fig. 2*A*). Induced sites were slightly enriched among CpG islands in that a higher % of induced sites than non-induced sites resided in CpG islands (Wilcoxon tests, *P* < 0.01 for both FW-hypo and FW-hyper), while among FW-hypo sites induced sites were slightly more likely than non-induced sites to reside in promoter regions (*P* = 0.004) (supplementary fig. S4, Supplementary Material online).

Sites that were induced in either population, or that were induced only in one of the two populations, had elevated *π* compared with sites that were not induced in either population (paired *t*-tests, all *P* < 0.01), while the *π* of non-induced DMCs was closer to that of non-DMCs (fig. 5*C*). Furthermore, the gain in *π* in FW among FW-hypo sites appeared to be driven by sites that had gained inducibility (i.e., which were not ancestrally inducible), as this elevated *π* was observed among sites that were induced only in FW (paired *t*-test, *P* < 0.001), but not sites that were induced only in marine (*P* = 0.83). Meanwhile, among FW-hyper sites, *π* was consistently significantly lower in FW compared with marine (paired *t*-tests, *P* < 0.05), with the exception of sites induced only in FW in which the difference was not significant (*P* = 0.09). When induced sites in each population were assigned to ranks according to the mean absolute induced methylation change (i.e., regardless of whether salinity change induced lower or higher methylation), *π* increased linearly with the mean induced methylation change in both marine (linear model, *R*^2^ = 0.83, *P* < 0.001) and FW populations (*R*^2^ = 0.59, *P* < 0.001) (fig. 5*D*), suggesting that environmental inducibility can reliably predict nucleotide diversity.

Compared with non-induced sites, sites that were induced in either population had significantly elevated pairwise Fst in both FW-hypo (paired *t*-test, *P* < 0.001) and FW-hyper categories (*P* = 0.002) (fig. 5*E*). However, this elevated Fst was driven by sites that were induced in FW, as sites induced only in FW had significantly elevated Fst in both FW-hypo (*P* < 0.001) and FW-hyper categories (*P* = 0.008), while sites induced only in marine fish did not show elevated Fst compared with non-induced sites. Indeed, FW-hypo sites that were induced only in marine even had lower Fst than non-induced sites (*P* = 0.046). The elevated Fst of DMCs (as seen in fig. 2*B*) was therefore likely driven by sites that are inducible in the FW population. Finally, when induced sites in each population were ranked according to the degree of induced methylation change in that population, sites that were induced in the FW population showed a weak positive correlation between inducibility and Fst (linear model, *R*^2^ = 0.25, *P* < 0.001), while sites that were induced in the marine population did not show such a correlation (*R*^2^ = −0.01, *P* = 0.4) (fig. 5*F*).

## Discussion

Here, we examined the relationship between DNAme differences and nucleotide diversity in an ancestral and a derived population of wild three-spined stickleback. For this purpose, CpG sites with different methylation status in FW compared with the marine population were interpreted as changes in DNAme that occurred in the course of FW colonization. Our analyses show that genetic diversity is intimately linked to variation in DNAme across both long (population differentiation) and short timescales (environmental responses). This link between DNAme and nucleotide diversity can shed light on the evolutionary forces acting on methylation state and could hint at the extent to which epigenetic changes precede sequence evolution.

### Sites Prone to Methylation Divergence Have High Standing Genetic Variation Driven by Hypermutability of 5mC

Despite applying stringent filtering to retain only CpG sites that were detected in all RRBS individuals (requiring all individuals to be either C/C or heterozygous at reference CpG loci), we found that not only did DMCs harbor SNPs among the individuals represented in the pooled sequencing data set but also were even enriched for them (fig. 2*A* and *D*). The high nucleotide diversity of DMCs was regardless of population, indicating that DM occurred at sites of high standing genetic variation. Consistent with a probable past bottleneck (Terekhanova et al. 2014), nucleotide diversity was reduced in FW, a pattern that held for FW-hyper sites despite the expectation that sites gaining methylation should incur higher mutation rates (Xia et al. 2012). However, FW-hypo sites—those that had lost methylation in FW—exhibited a slight increase in *π* in FW compared with marine, implying relaxed selection among these sites in the derived population.

The use of various measures of genetic diversity in parallel can provide insights into possible evolutionary processes at a fine scale. DMCs had higher diversity than non-DMCs as measured by both *π* (fig. 2*A*) and *θ* (supplementary fig. S2, Supplementary Material online), indicating both more pairwise differences at polymorphic sites and more polymorphic sites overall among these sites. However, DMCs also had higher Tajima's *D* (higher proportion of intermediate-frequency alleles) than non-DMCs within the marine population (fig. 2*C*), suggesting that sites with a tendency to diverge in methylation state are those that were already under weaker selective constraint or possibly under balancing selection (Jackson et al. 2015). The generally higher Tajima's *D* in FW was expected following a recent population bottleneck (Stajich and Hahn 2005). However, this increase was not observed at FW-hyper sites, suggesting that although FW had an overall tendency to accumulate intermediate-frequency alleles, this was impeded at FW-hyper sites, possibly due to increased selective constraint. The elevated Fst (fig. 2*B*) of FW-hypo and FW-hyper sites compared with non-DMCs further suggests that both types of DMC are subject to some degree of differential selection.

DNAme and mutations rates are intrinsically linked by the hypermutability of 5mC. Indeed, we find that the patterns of nucleotide diversity are driven by higher abundance of C-T/G-A SNPs among DMCs, but not other SNP types (fig. 2*D*), suggesting that they are driven by higher rates of spontaneous deamination of methylated Cs (Xia et al. 2012). The hypermutability of 5mC may further explain why FW-hypo sites were >6× more common than FW-hyper sites. If sites that acquire methylation during the transition to a new environment are more prone to mutation, then many such methylation gains would be transient. Thus, stable gains in methylation would be more difficult to attain than stable losses, and active selection might be required for their maintenance. Indeed, as sites with newly gained methylation must escape deamination in several individuals in order to be detected in the DM analysis (due to stringent site filtering), FW-hyper sites may be enriched for the subset of new methylations that are under selective constraint.

We also note that while *π* was increased in FW relative to marine at FW-hypo sites (fig. 2*A***)**, this increase was not observed in *θ* (supplementary fig. S2, Supplementary Material online) or the percentage of C-T/G-A SNPs (fig. 2*D*), which instead showed similar values in marine and FW. Nevertheless, given the lower overall diversity of FW, this population would need to have incurred elevated mutation rates at FW-hypo sites in order to reach similar values of *θ* and C-T/G-A SNPs to the marine population. Therefore, the slightly elevated *π* in FW is likely to have been driven largely by accumulation of C-T/G-A SNPs.

Overall, contrasting the different measures of nucleotide diversity reveals complex patterns of sequence evolution at methylation sites, reflecting ancestral standing genetic variation and possibly recent changes in the fitness landscape of methylation sites.

### Relationships Between Nucleotide Diversity and Methylation Frequencies Further Imply Differential Selection of DMCs

The relative frequencies at which sites are methylated (expressed as mean{PM}; fig. 3) capture both intraindividual and interindividual variations in methylation state. Although the RRBS data derived from a specific tissue, gill, such a tissue is nevertheless heterogeneous, comprising of different specialized cell types (Pan et al. 2022). Very high or very low mean{PM} values are therefore likely to comprise sites where the same state is consistently maintained across the majority of cells and/or cell types. Although cell-type–specific methylation is likely to be important in some contexts (Loyfer et al. 2023), it could nevertheless be inferred that sites with consistent methylation state are more likely to be selectively constrained. We observed that among non-DMCs, most sites had either very high or very low mean{PM}, suggesting that most sites have a methylation state that is consistently maintained across cell types (i.e., consistently methylated or nonmethylated). Non-DMCs with intermediate mean{PM} had higher *π* than those with very high or very low mean{PM}, again indicating stronger selective constraints on sites that are consistently either methylated or nonmethylated. This is consistent with previous observations that sites in the human genome with low-to-intermediate methylation frequency in vitro have higher mutation rates in human populations (Xia et al. 2012). We found that in sticklebacks, DM in the FW population was characterized by shifts either towards (FW-hypo) or away from (FW-hyper) intermediate mean{PM} (fig. 3*A*), corresponding with increase or decrease in *π*, respectively (fig. 3*B*). Accordingly, *π* increased in FW with the degree of hypomethylation and decreased with the degree of hypermethylation, while Fst tended to increase with the degree of difference in either direction (fig. 3*D* and *E*). Combined, these patterns imply that DM occurs alongside differential selection, in that (stable) gains in methylation tend to be selectively constrained while sites that lose methylation are released from selective constraint. This would make sense given that a loss of methylation relinquishes the requirement of the locus to remain as a CpG dinucleotide.

### Genetic Variation Reflects (Ancestral) Epigenetic Variation

Interindividual variability of DNAme remains understudied in natural populations, and yet it may hint at the processes by which the methylome evolves. Using the same RRBS data set, Artemov et al. (2017) previously showed that methylation was more variable in the derived FW population. Here, we show that this effect depends on DM (fig. 4*A*). Indeed, the higher variability in FW appeared to be driven largely by FW-hypo sites that showed a Gaussian-like distribution of SD_meth_ values in the FW population compared with a strongly left-skewed distribution of the same sites in marine. FW-hyper sites on the other hand became less variable in FW. Loss of methylation in FW therefore appears to be characterized by relaxed control of methylation state, while gain of methylation is associated with tighter control. Indeed, heightened variability of FW-hypo sites suggests that loosening of regulation is itself the cause of methylation loss.

*π* tended to increase with variability of methylation (fig. 4*B*), which would support the hypothesis that sites with less tightly maintained methylation state are under weaker selective constraint. This pattern was consistent across all three site categories in the marine population but was absent among FW-hypo sites in FW. Genetic variation therefore reflects ancestral but not recently acquired variability in methylation state. Relaxed selection on sites that lose methylation would lead to an accumulation of C-T mutations, while stronger selective constraint would reduce the nucleotide diversity of sites with stable methylation gain. Concordantly, sites that became less variable in FW tended to lose *π*, while sites that became more variable tended to gain *π* (fig. 4*D*). The pattern was more prominent among FW-hyper sites that predominantly had both decreased SD_meth_ of methylation and decreased *π*. Among FW-hypo sites, the lack of correlation between SD_meth_ and *π* in FW (or difference in SD_meth_ and difference in *π*) could be explained by the relatively young age of the FW population (∼700 years) and subsequent lack of time for mutations to accumulate.

Although it is plausible that the increased *π* and SD_meth_ of FW-hypo sites reflect relaxed selection on the regulation of methylation state, an alternative hypothesis is that variable loss of methylation reflects epigenetic responses that have occurred only in a fraction of individuals in the population. This scenario would also be consistent with elevated *π* of the FW-hypo sites; if these differential epigenetic responses were genotype-specific, the elevated *π* would reflect standing genetic variation as opposed to new mutations. Such a scenario would be consistent with a soft sweep (Hermisson and Pennings 2017), in which selection could act on many different genetic and epigenetic loci, thus maintaining diversity at both levels.

### Environmental Inducibility of DNAme May Predict Sequence Evolution

The potential importance of epigenetic mechanisms in mediating plastic responses has long been discussed (Johnson and Tricker 2010) and, more recently, demonstrated experimentally (Stajic et al. 2019). Although the environmental induction of a particular epigenetic state (e.g., addition or removal of DNAme) can occur in the context of adaptive mechanisms (Lämke and Bäurle 2017), such an induction may not necessarily constitute an adaptive response (see also Hu and Barrett 2023). As such, we considered environmental inducibility in a different context, in that the degree of environmental inducibility of methylation state is (inversely) indicative of the degree of intrinsic regulation. We therefore use the term “inducibility” loosely to refer to the sensitivity of a site to methylation change in response to the environment, regardless of its potential adaptive importance. We found that elevated *π* of and Fst of DMCs was driven by sites that were environmentally inducible (fig. 5*A*, *B*, and *E*), further supporting a hypothesis of relaxed regulation and relaxed selective constraint at sites that are responsive to environmental conditions. Furthermore, the increased *π* among FW-hypo sites in FW relative to marine was driven by sites that were induced only in FW, that is, those not induced in the ancestral population, suggesting that nucleotide diversity is more likely to accumulate at sites where intrinsic control of methylation is relaxed (and therefore more sensitive to the environment). Indeed, the positive correlation between *π* and the degree of inducibility (fig. 5*C*) suggests that the more sensitive the methylation state is to the environment, the more likely mutations are to be selectively neutral. Therefore, shifts in inducibility (in addition to shifts in methylation variance, as discussed above) may precede shifts in nucleotide diversity. Our results suggest that the majority of environmentally inducible sites are simply “blowing in the wind” and do not have important functions for plasticity that would constrain nucleotide diversity. Nevertheless, in their analysis of Baltic Sea sticklebacks, Heckwolf et al. (2020) observed that the Fst of induced sites (marine fish responsive to lower salinity) depended on the direction of the induced change. Sites that were induced to the “evolved” methylation state observed in the derived FW population had lower Fst than those that were induced in the opposite direction. This suggests that some environmentally inducible sites are indeed constrained by selection due to the importance of site plasticity. Here, we did not consider the direction of inducible change, merely considering inducibility as a proxy for the relative weakness of intrinsic regulation.

Again, these observations could also be reconciled with the scenario of a soft sweep, as it is also possible that plasticity of only some methylation sites is necessary to confer adaptation. In other words, plasticity of multiple sites provides multiple alternate routes to adaptation. As many methylation sites would therefore be redundant, they could be lost to mutation without detrimentally affecting the organism's capacity for adaptive plasticity.

### Limitations and Future Directions

Our analyses have revealed striking associations between genetic and epigenetic variation in divergent stickleback populations. However, we must acknowledge limitations including the extent of the data used to address the question, technical and analytical limitations, and knowledge gaps that pave the way for future investigations.

A key limitation is that the RRBS data we used came from only a single tissue type (gill), and therefore we were unable to determine which methylation differences between populations are tissue specific and which are organism wide. However, many divergent methylation states are not tissue specific, as a recent analysis of divergent cichlid ecotypes showed that a high proportion of DMRs were shared across tissue types (Vernaz et al. 2021). Also, with respect to the studied divergence between marine and FW environments, gills are key to salt homeostasis and their ability to respond to changes in osmolarity affects the entire organism. A similar sampling limitation is that, while the two data sets used in this study included marine fish collected from similar locations in the White Sea, we cannot be sure that pool-seq marine and RRBS marine individuals were representative of the same population. In a structured population, DNAme and nucleotide variation may covary at different sites across different branches of the population. This would cause some relationships to be missed if RRBS and pool-seq individuals came from different branches of the population.

The detection of DM is highly sensitive to the analytical methods applied. Firstly, the use of RRBS instead of whole-genome BS-seq limited the number of sites that could be analyzed to ∼6.9% of genome-wide CpGs. It is also selective for CG-rich regions, and as such approximately half of the analyzed sites belonged to CpG islands (see supplementary fig. S3, Supplementary Material online). Therefore, the patterns we observe may not necessarily be representative of genome-wide patterns. RRBS remains however a powerful and cost-effective means of methylome interrogation (see also Klughammer et al. 2023). Secondly, the retention of individual-specific CpG sites can lead to the detection of DM simply due to differences in the abundance of CpGs available to be methylated—that is, directly due to SNPs (Wulfridge et al. 2019). We suggest that whether or not individual-specific CpGs are retained in an analysis, and by extension the definition of “differential methylation,” should depend on the goals of the study. Here, by excluding individual-specific sites, we aimed to detect DM that arose through the differential action of the methylation machinery and not due directly to nucleotide variation at the sites themselves. We acknowledge that excluding individual-specific CpGs risks ignoring a potentially high proportion of methylation variation and, although it was not the goal of this study to extensively characterize this variation, the results should be interpreted with this in mind. A third important analytical limitation stems from the necessity to filter C-T/G-A SNPs from BS-seq data to avoid A bases resulting from these SNPs being misclassed as unmethylated Cs that were bisulfite converted to Ts. Although we used a combination of three SNP callers designed for BS-seq data (see Materials and Methods), we cannot be certain that some DM was not the result of SNPs that these algorithms failed to detect (see Lindner et al. 2022).

In a broader context, our study is limited in that we only examined one population pair. It is therefore currently not known whether the patterns we observed occur more broadly across different local adaptations (in stickleback and other species) or whether they are idiosyncratic to the relatively recent colonization event considered in this study (∼700 years). The existence of far older populations, such as those in the Japanese archipelago that are estimated to have colonized FW ∼170,000 years ago (Kakioka et al. 2020), raises the question as to the fate of DM over longer periods. Over time, for example, the initially heightened methylation variance may return to a less variable state due to refinement of methylation states via selection or the removal of the CpG sites via accumulation of C-T transitions. Alternatively, no substantial accumulation of mutations over time would suggest that the heightened diversity of FW-hypo sites reflects standing genetic variation.

If, indeed, heightened methylation variance arises due to relaxed control of methylation state, the mechanisms by which this could occur are not known. Artemov et al. (2017) suggested that mutations in genes encoding epigenetic regulators may underlie increased methylation variance but did not identify any known epigenetic regulators in the vicinity of genomic regions differentiating marine and FW populations in the White Sea region. *Trans*- and *cis*-meQTL have however been identified in stickleback (Hu et al. 2021), some of which are indeed in the vicinity of genomic regions of high Fst between marine and FW populations. Differential selection on *trans*-meQTL in particular could have knock on effects on methylation sites across the genome.

Although our study considered only genetic variation in the form of SNPs at CpG sites themselves, DNAme is associated with other types of genome sequence alterations. These include mutations in non-CpG context (Walser and Furano 2010), recombination rate variation (Mirouze et al. 2012), and structural variation including copy number and transposable element variation (Guerrero-Bosagna 2020). Indeed, the role of structural variation in local adaptation is increasingly appreciated, and major inversions, transposable elements, and copy number variants are all proposed to have played a role in stickleback FW adaptation (Reid et al. 2021). The interplay between epigenetic variation and other forms of genetic variation therefore warrants further interrogation in the local adaptation context (see however Kim et al. 2015; Huang and Chain 2021).

Finally, although shifts in nucleotide diversity in certain methylation contexts may signify changes in the fitness landscape of methylation sites, how they translate to fitness itself at the organism-level remains to be elucidated. Exploring the influence of methylation site diversity on gene expression variation would be a step towards addressing the possible fitness consequences.

## Conclusions

By intersecting genetic and epigenetic data from naturally diverging populations, we have identified signatures of differential selection on DNAme sites that, combined with patterns of methylation variance and environmental inducibility, support a hypothesis that DM is driven by shifts in the degree of intrinsic control of methylation state in a derived population. Shifts in this control seem to precede increases in nucleotide diversity and may therefore indicate parts of the genome where diversification is imminent. Heightened diversity of DMCs may also reflect a soft sweep that retains diversity at both genetic and epigenetic levels, a scenario compatible with previous genetic studies of local adaptation in stickleback (Terekhanova et al. 2014; Roberts Kingman et al. 2021). Indeed, our results support the idea that epigenetic variation should be incorporated alongside genetic mechanisms of adaptation in models of species adaptation and evolutionary potential (Bernatchez 2016). Our analyses demonstrate the exciting potential held in published data sets for exploring combined patterns of genome and epigenome evolution. Further investigation is now required to evaluate the broader role of methylome variation in shaping genomic landscapes across populations and species and ultimately the influence of these interactions on fitness at the phenotypic level.

## Materials and Methods

### Data Sets

We obtained a RRBS data set published by Artemov et al. (2017) (Sequence Read Archive [SRA] project accession: PRJNA324599) comprising a total of six marine sticklebacks (of which three were exposed to lower salinity) and six FW sticklebacks (of which three were exposed to higher salinity; however, one of these three samples was excluded due to incomplete bisulfite conversion). The FW fish used for RRBS were sampled from Lake Mashinnoye, while the marine fish were sampled from the Kandalaksha Gulf. FW fish were also collected for pool-seq by Terekhanova et al. (2014) from Lake Mashinnoye (SRA run accession: SRR869609), while marine fish were collected from the Kandalaksha Gulf as part of the 2014 study and a subsequent 2019 study (Terekhanova et al. 2019). We selected the “White Sea, WSBS” sample from Terekhanova et al. (2019) (SRR7470095) as the marine sample for our comparison, given that it has a similar pool size to the Mashinnoye sample (12 vs. 10) and a similar number of 100 bp paired reads (64,176,648 vs. 62,016,859 after quality trimming). Sequence files were obtained in FASTQ format from the SRA and European Nucleotide Archive (ENA).

### Data Processing: RRBS

Raw RRBS reads were trimmed using TrimGalore v0.6.6 using default settings. Alignment to the Three-spined stickleback v.5 assembly (Nath et al. 2021) and subsequent methylation calling were carried out using Bismark v0.22.3 (Krueger and Andrews 2011) with Bowtie2 v2.3.4.1 as the aligner (Langmead and Salzberg 2012). Methylation calls were not strand specific. To remove sites harboring C-T/G-A SNPs that otherwise contribute erroneous counts of nonmethylated Cs, we ran three SNP callers on each sample: BS-SNPer v1.1 (Gao et al. 2015), Biscuit v0.3.14 (https://github.com/zhou-lab/biscuit), and CGmap-tools v0.1.2 (Guo et al. 2018). We then compiled the coordinates of all sites harboring C-T/G-A SNPs detected in any of the individuals by any of the SNP callers (either homozygous or heterozygous) and removed these sites from the Bismark coverage files containing the methylation counts (counts of Cs and Ts at each position). This approach detected 75% of C-T/G-A that were detected at high frequency in the FW pool-seq sample (supplementary fig. S5, Supplementary Material online). Further details of SNP calling from RRBS are provided in the supplementary methods, Supplementary Material online.

### Data Processing: Pool-Seq

Raw reads were trimmed with Trimmomatic v0.36 (Bolger et al. 2014) with the option SLIDINGWINDOW:4:20 and otherwise default parameters. Only reads that remained paired after trimming were kept. Reads were mapped to the Three-spined stickleback v.5 assembly with Bowtie2 v2.3.4.1 with default parameters (Langmead and Salzberg 2012). Sambamba v0.7.1 (Tarasov et al. 2015) was used to filter the alignments to retain those with MAPQ ≥ 20 and to remove PCR duplicates. This resulted in 46,588,899 and 35,691,831 high-quality alignments from marine and FW samples, equating to average genome coverage of 10.4× and 8×, respectively. SAMtools v0.1.18 (Danecek et al. 2021) was used to generate a pileup file from each BAM file, as required for the Popoolation and Popoolation2 toolkits.

### Identification of Differentially Methylated Sites in CpG Context (DMCs) and Subsampling of Non-DMCs

Site-level DM analysis was carried out using the methylKit R package v1.22.0 (Akalin et al. 2012), inputting the SNP-filtered Bismark coverage files. We omitted sites that did not have at least 5× coverage in each of the 11 samples as well as sites located on the mitochondrial chromosome and two sex chromosomes (chromosomes XIX and Y). We then filtered out sites that had either 0% or 100% methylation (i.e., no variation) in all samples from the main population comparison (3× marine and 3× FW). Three DM analyses were then performed separately, comprising the comparisons also described in Artemov et al (2017): 1) marine fish in saltwater versus FW fish in FW (main population comparison), 2) marine fish in saltwater versus marine fish in FW (marine low salinity treatment), and 3) FW fish in FW versus FW fish in saltwater (FW high salinity treatment). All groups comprised *n* = 3 with the exception of FW fish in saltwater (*n* = 2, due to low bisulfite conversion efficiency of sample SRR3632642). Regardless, we considered sites to be differentially methylated given a difference in percentage methylation of ≥15 and a false discovery rate (FDR)-corrected *P*-value of ≤0.05. The purpose of the experimental comparisons was to identify which population DMCs were also induced by salinity change, and so we did not consider sites that were induced by salinity change but not differentially methylated between populations. We also did not consider the direction of induced change (hypomethylated or hypermethylated in response to salinity change). Subsequently, we detected 91,320 sites that were FW-hypo compared with marine (of which 10,289 were induced in either population) and 14,508 sites that were FW-hyper compared with marine (of which 5,685 were induced in either population). A total of 895,121 sites were not differentially methylated between populations, a subsample of which we would use as reference sites when examining nucleotide diversity. Due to the possibility that DMCs could be distributed nonrandomly across a chromosome and given that nucleotide diversity can vary across a chromosome (e.g., lower diversity in centromeric regions), we used a sampling procedure which randomly selected one nondifferentially methylated site that was within 2-kb upstream or 2-kb downstream of each DMC. After removing duplicate samples, this resulted in a subsample of 99,585 nondifferentially methylated sites in CpG context (non-DMCs).

### Nucleotide Diversity of Site Categories

We used the variance-at-position.pl script from the Popoolation toolkit (Kofler, Orozco-terWengel, et al. 2011) to calculate within-population nucleotide diversity statistics (*π*, Watterson's *θ*, and Tajima's *D*) for different categories of site (e.g., non-DMC, FW-hypo, and FW-hyper) on each chromosome for each population. The decision to obtain estimates of *π* on a per-chromosome basis was because Popoolation's estimates of *π* are accurate over large numbers of sites but not at the single site level (Kofler, Orozco-terWengel, et al. 2011). Each site was labeled with its chromosome and its category within the analysis (e.g., chrI FW-hypo), and the labeled category was entered as the “gene ID” in a GTF file, such that variance-at-position.pl, which was developed to calculate diversity statistics per gene, was instructed to calculate *π* for each combination of chromosome and site category. A similar procedure was used to obtain *π* for sites ranked according to (difference in) mean{PM}, (difference in) SD_meth_, and absolute inducibility, whereby ranks were assigned using the bin() function from the OneR package, specifying 50 ranks each time. Sites were then labeled in the GTF according to their rank (regardless of chromosome), such that a single value of *π* was obtained for each rank. Variance-at-position.pl from Popoolation was run with the parameters –min-qual 20, –min-coverage 3, and –min-count 2. The majority of sites met the requisite 3× coverage for inclusion in nucleotide diversity estimates of marine (99%) and FW (93%). For the analysis of nucleotide diversity as a function of absolute inducibility, one rank was excluded from the FW population due to insufficient coverage (<60% of sites with 3× coverage).

Fst for different categories of methylation sites (including ranked sites) was obtained using the Popoolation2 toolkit (Kofler, Pandey, et al. 2011). “Gene-wise”.sync files were obtained from the pileup files using coordinates in the abovementioned gtf files and were used as input for the “fst-sliding.pl” script that was run with parameters –min-count 2, –min-coverage 3, –pool-size 22, –min-covered-fraction 0, –max-coverage 1,000, –window-size 1,000,000, and –step-size 1,000,000.

### Percentage of Sites With SNPs

To obtain the % of sites within each result category (non-DMC, FW-hypo, and FW-hyper) harboring SNPs of different types (C-T/G-A and non-C-T/G-A) in the pool-seq samples, we filtered the BAM files of each population to retain alignments corresponding with the positions of interest. We then ran GATK HaplotypeCaller (McKenna et al. 2010) with the –sample-ploidy set to the pool size 2× (24 for marine and 20 for FW) and otherwise default settings. The subsequent VCF file was then filtered using BCTtools v1.10 to retain only biallelic SNPs at the sites of interest. We subsequently extracted from the VCF a list of reference and alternate alleles at sites of interest harboring biallelic SNPs. We were therefore able to assign SNPs as either “C-T/G-A” or “other” and calculate the percentage of sites in each result category harboring biallelic SNPs of one of those two classes.

### Statistical Comparisons

All plotting and statistical analyses were carried out in R version 4.2.0 (R Development Core Team 2011), with plots generated using the ggplot2 package (Wickham 2011). For comparing nucleotide diversity between populations, we first used Shapiro–Wilk tests to determine whether the distribution of pairwise differences (paired chromosomes) differed significantly from a normal distribution. Paired *t*-tests were used in the case of normally distributed pairwise differences, and paired Wilcoxon tests were used in the case of nonnormally distributed pairwise differences. Linear models of nucleotide diversity parameters as a function of inducibility were fit using the lm() function from the stats package.

## Supplementary Material

msad068_Supplementary_Data

## Data Availability

This work used entirely preexisting data sets available under SRA project accessions PRJNA324599 (RRBS data), PRJNA204958, and PRJNA479509 (whole-genome pool-seq). Code for performing analyses has been deposited on GitHub in the repository: https://github.com/jamesord/StickMethDiv .
