# Supplementary material for: High Nucleotide Diversity Accompanies Differential DNA Methylation in Naturally Diverging Populations
Source: Mol Biol Evol. 2023 Mar 22;40(4):msad068. doi: 10.1093/molbev/msad068 (PMC10139703; doi:10.1093/molbev/msad068)
Supplement: msad068_Supplementary_Data [file msad068_supplementary_data.pdf]

# Supplementary material: High nucleotide diversity accompanies differential DNA methylation in naturally diverging populations

James Ord[1,2]*, Toni I. Gossmann[3] & Irene Adrian-Kalchhauser[1]

[1] Institute for Fish and Wildlife Health, University of Bern, Länggassstrasse 122, 3012 Bern, Switzerland

[2] Faculty of Biological and Environmental Sciences, University of Helsinki, Viikinkaari 1, 00790 Helsinki, Finland

[3] Computational Systems Biology, Faculty of Biochemical and Chemical Engineering, TU Dortmund University, Emil-Figge-Str. 66, 44227 Dortmund, Germany

Corresponding author: jms.ord18@gmail.com

## Supplementary figures

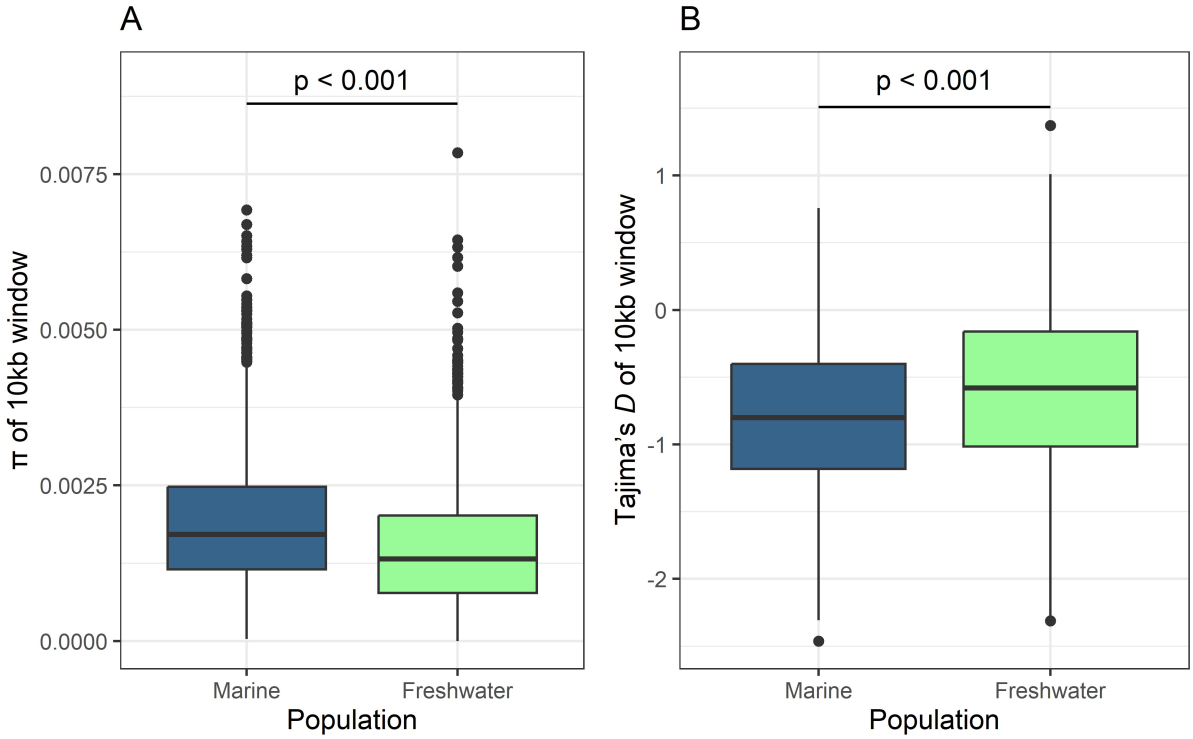


**Figure S1. Nucleotide diversity statistics of sliding windows across chromosome I of marine and freshwater population.** Estimates of π and Tajima's *D* in 5,924 10kb windows across chromosome I were derived from pool-seq data of marine (blue) and freshwater (green) populations. *P*-values represent results of general independence tests with approximated (Monte Carlo) null distributions and one-sided hypotheses.

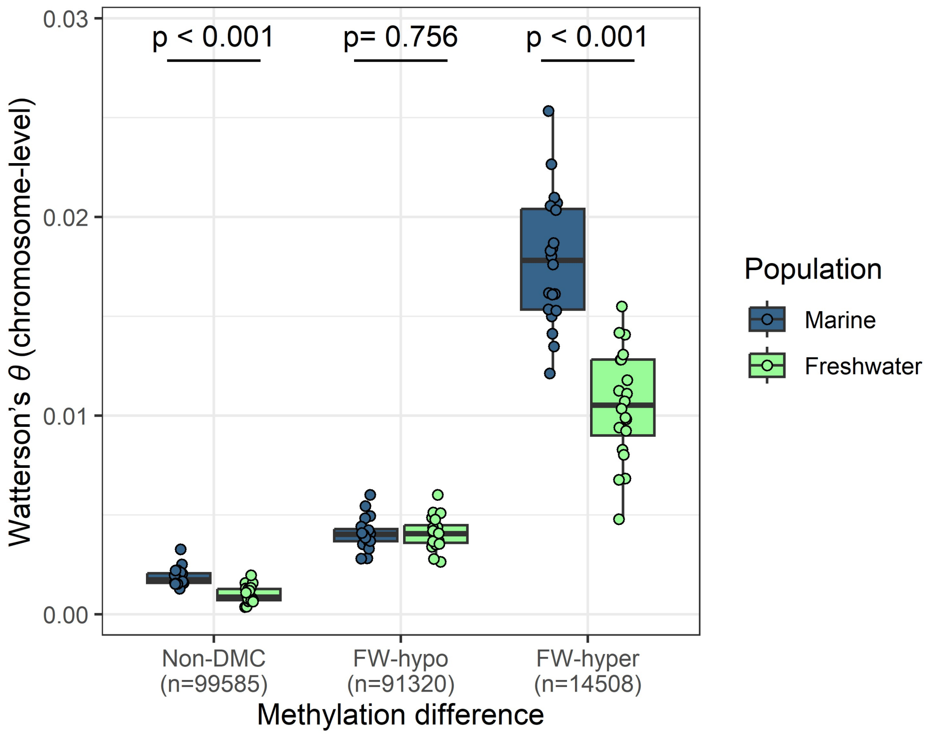


**Figure S2. Watterson's $\theta$ estimated from pool-seq of marine and freshwater sticklebacks for three classes of methylation site.** Sites were classified according to the direction of methylation difference in freshwater fish compared to marine. $\theta$ was estimated for each chromosome separately, such that one point represents an estimate from one chromosome. *P*-values derive from paired Wilcoxon tests (comparison of chromosome pairs).

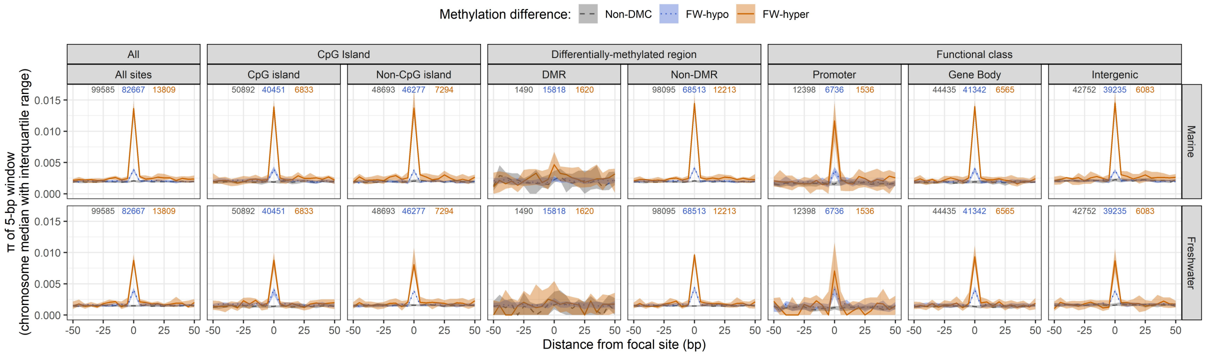


**Figure S3. Nucleotide diversity surrounding different classes of methylation site.** π was calculated in windows of 5bp with steps of 5bp extending 50bp either side of the focal site. Sites are classified according to the direction of methylation difference in freshwater fish compared to marine (non-DMC, dashed black lines; FW-hypo, blue dotted lines; FW-hyper, solid orange lines). Subplots are divided by population (marine, top and freshwater, bottom) and the category of genomic feature in which sites are considered (from left to right: all sites, sites within CpG islands, not within CpG islands, within differentially methylated regions (DMRs), not within DMRs, within promoter regions, within gene bodies, and within intergenic regions). Separate sets of sliding windows were derived for each chromosome and therefore the middle lines and ribbons denote the median and interquartile range of all chromosome-level estimates, respectively. The total numbers of non-DMC, FW-hypo sites, and FW-hyper sites considered in each category (total across all chromosomes) are shown at the top of each panel in grey, blue, and orange, respectively.

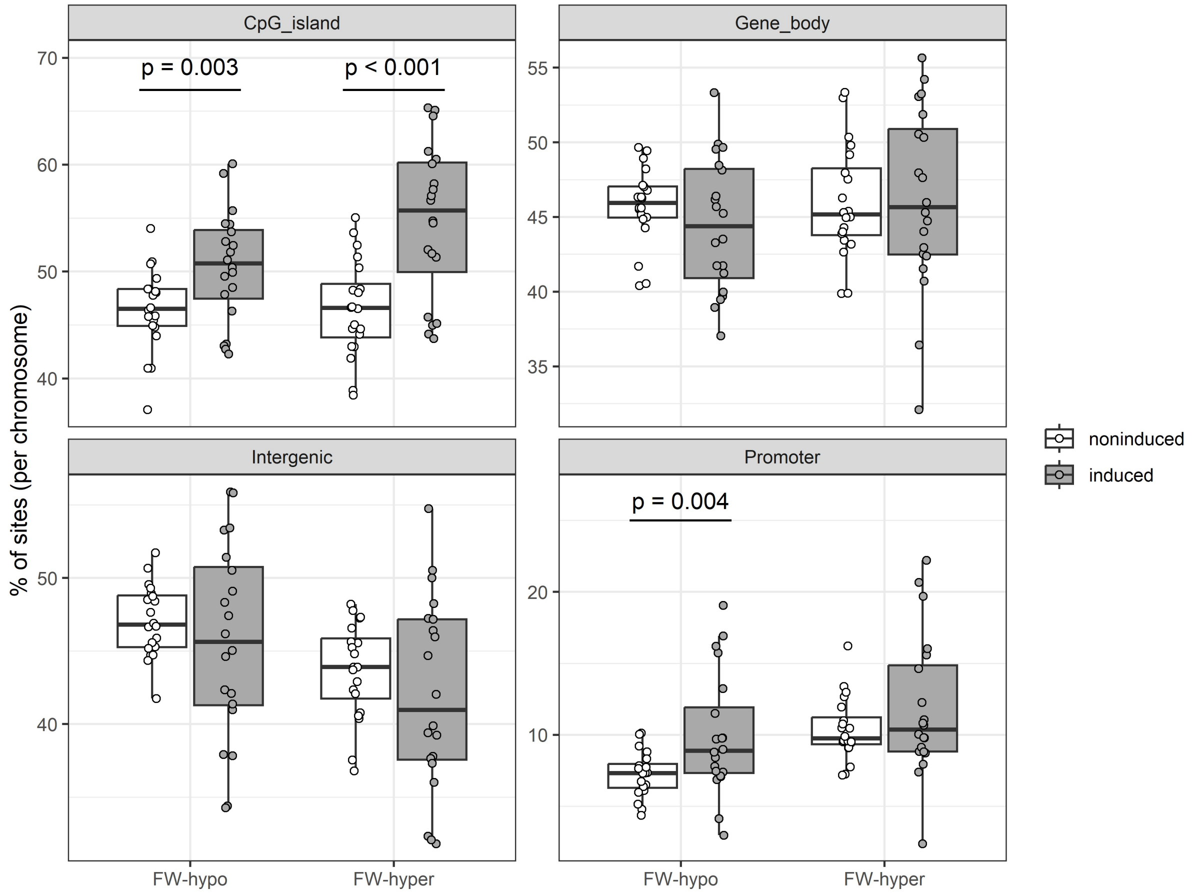


**Figure S4. Percentages of non-induced (white) and induced (grey) DMCs in FW-hyper and FW-hypo categories overlapping with different genomic features.** Each point represents the % of overlapping sites on one chromosome. *P*-values derived from paired Wilcoxon tests.

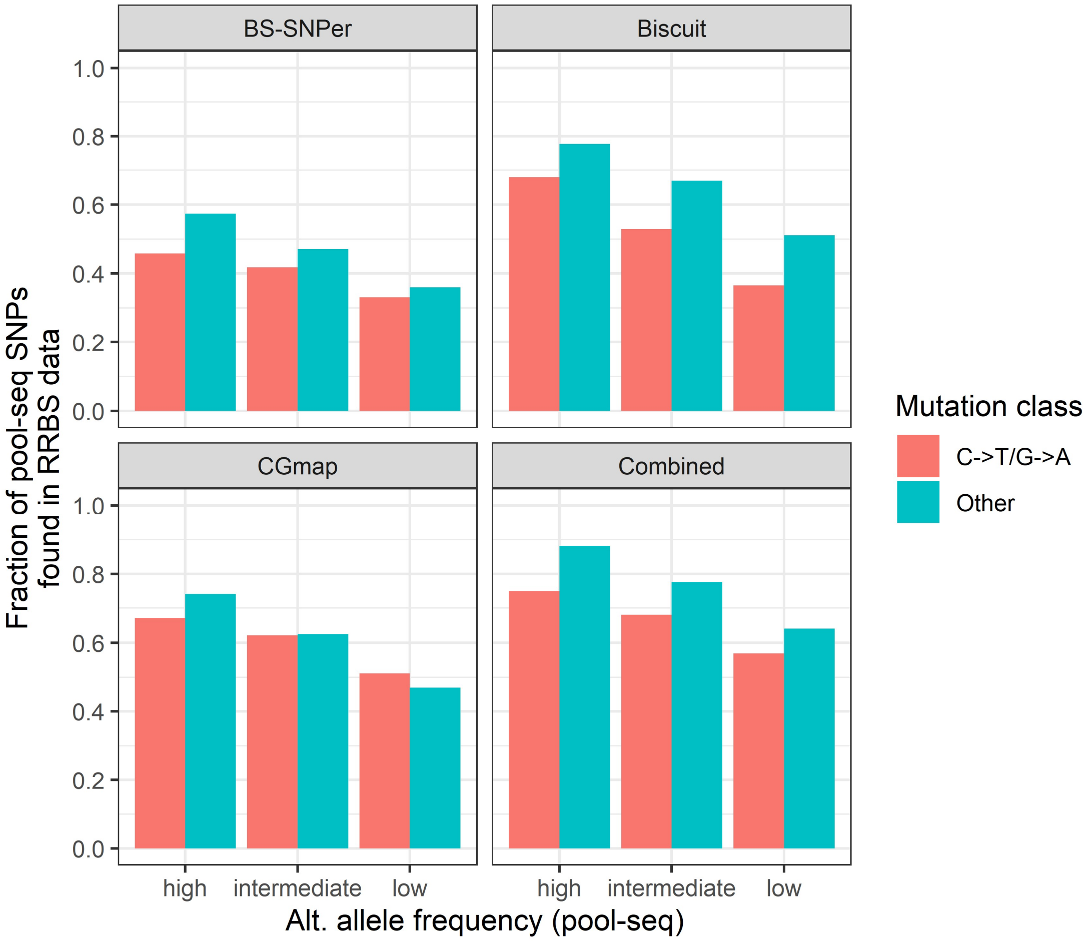


**Figure S5. Overlap of SNPs detected in RRBS individuals with biallelic SNPs detected at three different alt. allele frequencies in a freshwater population profiled by pool-seq.** The proportion of reads supporting the alt. allele in pool-seq was used as a proxy for alt. allele frequency (‘high’ = alt. allele supported by at least 80% of reads, ‘intermediate’ = alt. allele supported by < 80% >= 20% of reads, ‘low’ = alt. allele supported by <20% of reads). For each alt. allele frequency the proportion of pool-seq biallelic SNPs (C-T/G-A or other) that were also detected in any of 11 RRBS individuals by three SNP-callers (BS-SNPer, Biscuit, and CGmap) is shown, as well as the proportions detected by the combination of all three SNP-callers.

## Supplementary methods

*Sliding windows along chromosome I*

To obtain estimates of π and Tajima's *D* for each population along chromosome I, the pileup files generated from the WSBS and Mashinnoye pool-seq alignments were subset to include only data from chromosome I alignments. The variance-sliding.pl script from Popoolation (Kofler et al. 2011) was then run on each of the aforementioned pileup files. Parameters used for π estimation were: --window-size 10000 --step-size 10000 --min-count 2 --min-coverage 4 --min-qual 20. Parameters used for Tajima's *D* estimation were: -window-size 10000 --step-size 10000 --min-count 1 --min-coverage 3 --min-qual 20 --dissable-corrections on.

*Sliding windows around sites within different genomic features*

To visualise π in windows around DMCs in different genomic features, we first obtained the coordinates of genes (from the gff3 annotation file provided at https://stickleback.genetics.uga.edu), promoters (classified arbitrarily as the region spanning from 1kb upstream to 0.5kb downstream of the gene start; Heckwolf et al., 2020), CpG islands, and differentially methylated regions (DMRs). CpG islands were identified from the stickleback v.5 assembly (Nath et al. 2021) by running CpGIscan (https://github.com/jzuoyi/cpgiscan) with default parameters. DMRs (differentially methylated regions between marine and freshwater fish) were identified from the SNP-filtered Bismark coverage using the R package bsseq v1.32.0 (Hansen et al. 2012). Loci were retained for DMR calling if they had at least 5x coverage in each of the six samples (3x marine and 3x freshwater), and a region was considered differentially methylated given a T-stat of >/= 1.5 and a mean difference in % methylation of >/= 15. Subsequently, the coordinates of all aforementioned genomic features were compiled as BED files, such that for each feature type, a BED file containing the sites of interest (DMCs plus the subsample of non-DMCs) could be filtered using bedtools-intersect from bedtools v2.29.2 (Quinlan and Hall 2010) to return a list of the sites that were within that feature. We subsequently obtained lists of sites assigned to eight feature categories: 'all sites' (regardless of feature), 'CpG islands', 'non-CpG islands', 'DMRs', 'non-DMRs', 'promoters', 'genes', and 'intergenic'. We considered 'intergenic' sites to be any sites that did not overlap with either promoters or genes. For each site within each feature, we established a 5bp window centred on the site which was labelled as 0, and ten 5bp windows in steps of 5bp each side of the site labelled from -50 to 50. Window labels were then entered into GTF files for use with variance-at-position.pl from Popoolation (Kofler et al. 2011), such that a single π value was calculated for each window, encompassing all of the sites in a given category. The above was carried out separately for each combination of result category (non-DMC, FW-hypo, and FW-hyper), genomic feature category, and chromosome.

*Detection of SNPs in RRBS data*

To detect SNPs in RRBS data, we ran three SNP-callers on each sample: BS-SNPer v1.1 (Gao et al. 2015), Biscuit v0.3.14 (https://github.com/zhou-lab/biscuit), and CGmap-tools v0.1.2 (Guo et al. 2018). Bis-SNPer was run using a minimum coverage of 5 and otherwise default settings with Bismark-derived BAM files. Biscuit was run with default settings and with BAM files generated by the Biscuit aligner. CGmap-tools was run in binomial mode and otherwise default settings with Bismark-derived BAM files. We then compiled the coordinates of all sites harbouring C-T/G-A SNPs detected in any of the individuals by any of the SNP-callers (either homo- or heterozygous).

To test the efficacy of the SNP-calling approach, we examined the proportion of SNPs detected in one pool-seq sample that were also detected in at least one of the 11 RRBS individuals (marine or FW). We opted to use the FW pool-seq sample as a reference, as given its lower diversity it was considered less likely than marine to contain SNPs that are not present in the RRBS individuals. SNPs were called from pool-seq alignments using GATK HaplotypeCaller (McKenna et al. 2010) with the –sample-ploidy set to the pool size x 2 (24 for marine and 20 for freshwater), and otherwise default settings. The subsequent VCF file was then filtered using bcftools v1.10 to retain only biallelic SNPs. To facilitate comparison with RRBS, the reference SNP set was restricted to sites with at least 5x coverage in all RRBS individuals and the pool-seq sample. The reference pool-seq SNPs were classified according to the proportion of reads supporting the alternate / non-reference allele, which we took as a proxy for alternate allele frequency ('high' = alt. allele supported by at least 80% of reads, 'intermediate' = alt. allele supported by < 80% >= 20% of reads, 'low' = alt. allele supported by <20% of reads). The proportions of high, intermediate, and low-frequency pool-seq SNPs detected in the combined RRBS SNP set were 0.75, 0.68. and 0.6 for C-T/G-A SNPs and 0.88, 0.78, and 0.64 for other SNP types, respectively (Fig. S5).

While the 25% non-overlap of C-T/G-A SNPs may partly comprise false negatives, it may also be attributable to the relatively small numbers of individuals which limited the extent of nucleotide diversity that could be detected in both pool-seq and RRBS. Detection of a lower proportion of C-T/G-A SNPs compared to other SNPs may be partially due to greater difficulty in detecting these SNPs in BS-seq data (due to relying on read alignments from both strands). However, this may also be due to the higher rate of spontaneous C-T mutations meaning that a higher proportion of C-T/G-A SNPs comprise rare alleles (and therefore less likely than other SNPs to be detected in both pool-seq and RRBS).

## References


Gao S, Zou D, Mao L, Liu H, Song P, Chen Y, Zhao S, Gao C, Li X, Gao Z, et al. 2015. BS-SNPer: SNP calling in bisulfite-seq data. *Bioinformatics* 31:4006–4008.

Guo W, Zhu P, Pellegrini M, Zhang MQ, Wang X, Ni Z. 2018. CGmapTools improves the precision of heterozygous SNV calls and supports allele-specific methylation detection and visualization in bisulfite-sequencing data. *Bioinformatics* 34:381–387.

Hansen KD, Langmead B, Irizarry RA. 2012. BSmooth: from whole genome bisulfite sequencing reads to differentially methylated regions. *Genome Biol* 13:1–10.

Heckwolf MJ, Meyer BS, Häsler R, Höppner MP, Eizaguirre C, Reusch TBH. 2020. Two different epigenetic information channels in wild three-spined sticklebacks are involved in salinity adaptation. *Sci Adv* 6:eaaz1138.

Kofler R, Orozco-terWengel P, de Maio N, Pandey RV, Nolte V, Futschik A, Kosiol C, Schlötterer C. 2011. PoPoolation: A Toolbox for Population Genetic Analysis of Next Generation Sequencing Data from Pooled Individuals. *PLoS One* 6:e15925.

McKenna A, Hanna M, Banks E, Sivachenko A, Cibulskis K, Kernytsky A, Garimella K, Altshuler D, Gabriel S, Daly M, et al. 2010. The Genome Analysis Toolkit: A MapReduce framework for analyzing next-generation DNA sequencing data. *Genome Res* 20:1297–1303.

Nath S, Shaw DE, White MA. 2021. Improved contiguity of the threespine stickleback genome using long-read sequencing. *G3* 11: jkab007.

Quinlan AR, Hall IM. 2010. BEDTools: a flexible suite of utilities for comparing genomic features. *Bioinformatics* 26:841–842.
